# Strengthening the social resilience of people living at the intersection of precariousness and migration during pandemics: action recommendations developed in Munich, Germany

**DOI:** 10.3389/fpubh.2023.1201215

**Published:** 2023-08-02

**Authors:** Zeliha Asli Öcek, Mandy Geise, Anna-Maria Volkmann, Acelya Basili, Vera Klünder, Michaela Coenen

**Affiliations:** ^1^Institute for Medical Information Processing, Biometry, and Epidemiology (IBE), Chair for Public Health and Health Services Research, Medical Faculty, LMU Munich, Munich, Germany; ^2^Pettenkofer School of Public Health, Munich, Germany; ^3^Rachel Carson Centre for Environment and Society, LMU Munich, Munich, Germany; ^4^Netherlands Institute for Health Services Research, Utrecht, Netherlands; ^5^University College London, London, United Kingdom

**Keywords:** migrants, precariousness, social resilience, vulnerability, pandemics, COVID-19, action recommendation

## Abstract

**Introduction:**

An EU-funded project in five countries examined vulnerability mechanisms during the COVID-19 pandemic. The research team in Germany concentrated on people living at the intersection of migration and precariousness. The study aimed first to provide an understanding of how migrants living in precarious conditions in Munich had been affected by the pandemic, both from their own and from experts’ perspectives. The second aim was to develop action recommendations to reduce structural vulnerabilities and increase resilience with a view towards improved pandemic preparedness.

**Methods:**

The study followed a two-phase process. The first was a qualitative study based on interviews with 25 migrants and 13 experts. In the second, researchers developed action recommendations based on the vulnerability/ resilience factors that had been generated in the first phase. Three consecutive meetings with stakeholders (expert panel, focus group discussion with two migrant organization, meeting with the Munich Migration Council) were then held to further strengthen the draft recommendations.

**Results:**

Content analysis revealed twelve vulnerability and eight resilience factors in three domains (COVID-19 prevention; human rights, living and housing environment; social support). Migrants had limited access to COVID-19 prevention measures; living conditions made outbreaks inevitable; uncertainty about legal status, employment, and housing, as well as stigma and discrimination, exacerbated their precariousness; social support had decreased; and resilience mechanisms had failed. The initial draft of recommendations contained 24 proposed actions. The meetings added recommendations such as enhancing psychosocial support, preventing ghettoization, improving social housing, preventing the interruption of language education in times of crisis, severe penalties for media stigmatisation and proactive truth-telling. The final list included 30 actions.

**Conclusion:**

In Munich, the COVID-19 pandemic exacerbated vulnerability mechanisms commonly associated with being a migrant. The recommendations developed here speak to those vulnerabilities but need to be refined further to be more actionable and comprehensive. Nonetheless, the recommendations and the processes that led to them highlight the importance of migrant-inclusive approaches and empowerment in increasing migrants’ resilience to future crises.

## 1. Introduction

Crises such as pandemics have their most devastating effects on those whose lives are already characterized by insecurity (1, 2). A hazardous lack of security or stability, dependence (3) on uncertain developments, or dependence on the will of others is referred to as precariousness. The concept of precariousness encompasses components that interact synergistically such as income, employment, housing, access to food and the enjoyment of civil, economic, and social rights (3–10). All these components are the results of political, economic, and social structures that may generate injustice and inequalities among citizens (11) and that frequently weigh disproportionately heavy on the financial, job, housing, and food security of migrants (5, 7, 12, 13).

Alongside precariousness, the general term “vulnerability” has become frequently used in the discourse around migrant health and wellbeing (14). However, as noted by Atak et al. (15), what is meant by “migrants in vulnerable situations” is often ill-defined and the elasticity in the meaning of this term may lead one to discard the fact that the precariousness in which migrants find themselves is often constructed by states and other actors. In other words, it is not inherently linked to the status of being a “migrant.” Problematically, employing an unconsidered vulnerability narrative may serve to portray migrants as helpless victims, or as weak and without agency. The concept of “precariousness,” on the other hand, detaches the qualification from the person and indicates, we would argue, that much of the “vulnerability” of migrants is policy-driven (15). Also, according to Miller (11), precariousness captures a feature of the caregiving landscape that vulnerability does not quite address, namely, fundamental instability. However, the categorization of vulnerability based on its source eliminates this ambiguity. Temporary or permanent physical characteristics, such as disabilities, chronic illness, or indeed pregnancy, that are intrinsic to the human condition should be referred to as “inherent vulnerability.” Vulnerability arising from injustice in social, political, economic, or environmental structures, on the other hand, categorized as “situational” or “structural” (15). The latter category takes the social determinants of health perspective as a foundation, which includes domains such as financial security, residence, risk environments, access to food, social network, legal status, education, discrimination (16). This classification enables us to say that a person’s physical conditions render him or her inherently vulnerable, but if this person is shut out of society as a result of this condition and is unable to benefit from the opportunities that other people have, there is a structural issue. Distinguishing structural vulnerability and precariousness from inherent vulnerability allows drawing attention to the role of states and inequitable global migration systems (15, 17) and enhances the design and implementation of effective solutions for the protection of migrants (15).

Those whose lives are at greater risk of structural vulnerability are frequently also at risk of precariousness and may have fewer reserves to draw on, reducing their resilience (4). Resilience is the third and final term we look at closely in this introduction. Opposing the social-ecological resilience approach, which focuses on how individuals and communities adapt to external threats, authors such as McKee et al. (4) and Preston et al. (18) have argued for a “critical social resilience” approach. Such an approach addresses institutional inequalities and power relations that shape migrants’ capacities to obtain resources, especially during a crisis like the COVID-19 pandemic. Rather than seeing migration as an external threat, which risks eliciting anti-immigrant responses during the pandemic, critical social resilience proposes a move from an individualistic focus to a collective idea, emphasizing local and institutional support in newcomers’ efforts to build their lives, especially when faced with unexpected events such as a global pandemic, and recognising migrants as partners in shaping post-pandemic societies (4, 18).

The work presented here is the result of a multi-stage study carried out in collaboration with the *Sonar-Global Network* (19). The network coordinated a project involving five countries (Germany, Malta, Italy, France, and Slovenia) to investigate how various mechanisms that cause vulnerability or resilience have changed during the COVID-19 pandemic. Initial findings of the study in Munich, Germany, confirmed our expectation that migrants who were already living in precarious conditions and were vulnerable prior to the pandemic were the most affected by the crisis. As a result, the Munich research team concentrated on the question of how to strengthen this particular group’s resilience. It was decided to follow an approach that reveals the local situation by addressing the concepts of precariousness, structural vulnerability and social resilience from a global perspective and recognizes migrants as some of the key factors in building an equitable post-pandemic life. Accordingly, our study aimed to provide an understanding of how migrants living in precarious conditions in the Munich metropolitan area have been affected by the COVID-19 pandemic from both their own and from experts’ perspectives. Based on this understanding, we developed action recommendations that will plausibly reduce structural vulnerability and strengthen social resilience in times of crisis.

## 2. Materials and methods

**Figure 1:**
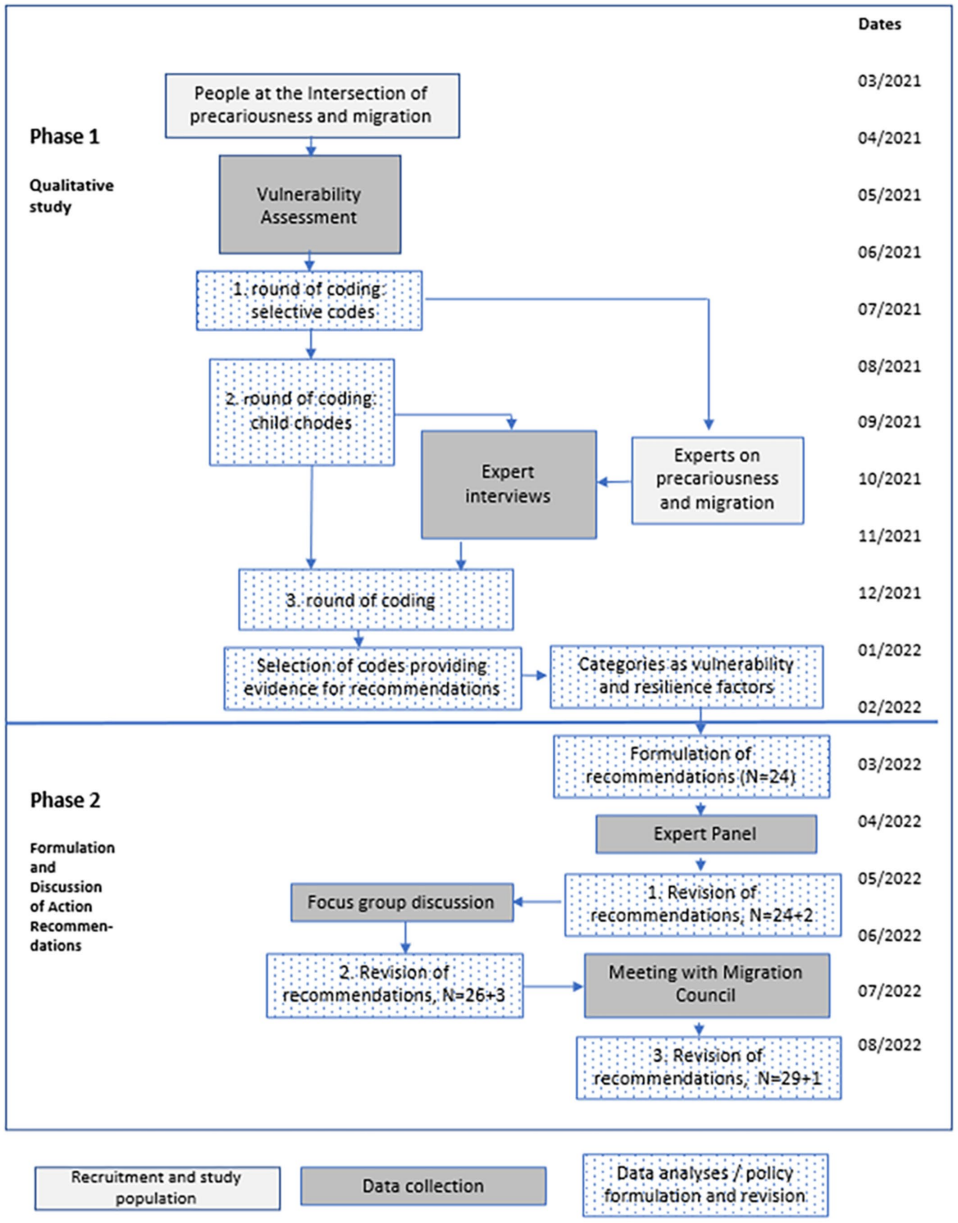
Development process of the action recommendations.

The study was performed in the metropolitan area of Munich (Germany) which is the capital city of the Federal State of Bavaria. The reason why Munich was selected for this study is that the Ludwig Maximilian University of Munich (LMU), Sonar-Global’s German project partner, is based there. Action recommendations were developed in a two-phase process (Figure 1). The first phase was a qualitative study defining vulnerability and resilience factors of immigrant people living in precarious conditions experienced during the COVID-19 pandemic. In the second phase, a set of action recommendations matching these factors was formulated and discussed with community representatives for further development.

### 2.1. Phase-1: qualitative study

#### 2.1.1. Recruitment and study population

Two groups of participants were consecutively included in the Phase-1. The first group consisted of people with various socio-economic disadvantages and physical impairments (such as living with chronic disease or disability) identified through a purposeful sampling method. Specifically trained fieldworkers were recruited who, through their networks, could reach out to different groups with those characteristics. Of the 82 people aged 18 and above reached in total, 25 (Table 1) were included in the present study because they came to Germany in the second half of their lives and met at least one of the following precariousness criteria (3–9).

1. Precarious housing: Severely unfavourable housing and its surroundings, insecure, unaffordable, and unstable housing. People living in refugee shelters, social welfare housing, flats shared with too many people they did not choose, and those who must move frequently due to very limited, or no tenancy agreements were considered to meet this criterion.
2. Precarious working: Low-skilled, low-paid, unsustainable, and insecure work, harsh working conditions. Workers without legal employment contracts, casual employees, temporary employees, and manual laborers are examples of precarious workers in the study population.
3. Resource insecurity: Severe economic insecurity, lack of social support, severe insecurity in terms of residence permit. Individuals who were struggling to meet their basic needs due to lack of financial means whose situation was unlikely to improve in the near future, as well as people whose refugee status in Germany had not yet been determined or who were awaiting a residence permit, preventing them from working or forecasting their future fulfilled this criterion.

**Table 1:** Characteristics of the study population – 2 (Experts at the intersection of precarity and migration).

Expert (E)	Migration back-ground	Explanations
E-1	Yes	Midwife with PhD thesis on access to maternity services among refugees. She is a member of an organization for refugees.
E-2	Yes	Professor in business administration. He is supporting immigrant families and small businesses. He is a highly respected person in his community.
E-3	Yes	Professor in care, manages of a centrum for people with disabilities. Many of her clients have a migrant background.
E-4	Yes	Social pedagogue in an organization where he provides legal advice on asylum and migration. He is the chairman of an initiative group for intercultural encounters and education.
E-5	Yes	Sociologist and president of an academy, which is the foundation of a confederation of Catholic organizations. She is in constant contact with social workers and migrants.
E-6	No	Physician at a rehabilitation clinic. Most of his patients are migrants.
E-7	Yes	She is the leader of an umbrella organization of 100 associations representing eighty nationalities in Munich.
E-8	Yes	Manager at a centre for new migrants from East Europe who are looking for work and the president of a migrant association with 14,000 members.
E-9	No	For 9 years he has been a volunteer for a charity organization with a focus on food. He is on the board of the Munich branch.
E-10	No	Pedagogue and the contact person for the Education and Science Union. He counsels students and represents students with severe disabilities
E-11	No	Chairman of the works council of an IT company where most of the employees are newly arrived migrants
E-12	No	Psychiatrist and president of a diaconal institution for people with mental illness. He has many years of experience with people living in precarious situations.
E-13	No	Psychotherapist, who is been working for an organization that aids in addiction support, rehabilitation, youth welfare. Most of his clients are in precarious conditions.

The second participant group consisted of a cohort of individuals working with vulnerable populations (Table 1). Those individuals were selected through internet search using search terms such as *income*, *discrimination*, *legal problem*, *housing*, *education*, *work*, *integration*, *language, and support*. A list was compiled of related committees, aid groups, and organizations in Munich, including academics, journalists, and healthcare workers. During their weekly meetings, the researchers evaluated the compliance of each potential participant with these criteria. Individuals who had at least 3 years of experience in migration issues in Germany through academic studies, professional or volunteer activities, and a solid understanding of the German language were eligible for the study. Names reached through snowballing techniques were added to this list. Emails were sent inviting individuals themselves or a representative of the organizations for individual interviews. Thirteen people were interviewed who will henceforth be referred to as “experts.” Experts had knowledge and professional experience on migration and precariousness.

For both groups, coding took place concurrently with the recruitment and data collection, and recruitment was stopped when data saturation was reached in the third stage of coding.

#### 2.1.2. Data collection

The first group was interviewed using a standardised Vulnerability Assessment tool (19), developed by one of the authors (AV) and adapted to examine how various mechanisms that cause vulnerability or resilience have changed during the COVID-19 pandemic in the Munich area. The same tool was also employed by the other four country members of the Sonar-Global Network participating in the study.

The tool was composed of a demographic questionnaire and a semi-structured interview guide. The questionnaire included questions regarding sociodemographic features, household characteristics (type of dwelling, presence of outside space, ownership of the house, persons living in the household), income and expenses (annual income, change of the income due to pandemic, any financial support, sufficiency of the income, healthcare insurance, burden of healthcare expenses) and employment (employment status, number of working days and hours, current job, place of work and the likelihood of a change in all of these within 6 months). These questions were used to assess whether participants met the required criteria to be described as living with precariousness. The semi-structured interview guide included questions about access to and use of COVID-19 information and protective measures as well as pandemic rules, support received from any official organization, source of any assistance received and social connections. Respondents were also asked about their perception on equality in their neighbourhood. Thematic axes of the interviews and related questions are presented in Table 2.

**Table 2:** Thematic axes of the interviews with people living at the intersection of precarity and migration and related questions.

Thematic Axes	Related questions
Support	Have you recently received any kind of help, support, or assistance from any public service or formal organization, whether health-related or not?
	Do you know of any public services or formal organizations that help people with COVID-19?
	In general, do you think that there is enough help, support, or assistance for people available where you live?
	Would you say that, in general, people in your community trust those who provide help, support, or assistance?
Neighbourhood	Do you feel integrated in your neighbourhood?
	Would you say that your neighbourhood is a healthy and safe place to live?
Social connections	Do you consider yourself part of a community?
	Do you personally feel socially well connected? Do you sometimes feel isolated or lonely?
Equality	Would you say that everyone living in your neighbourhood is treated equally? Is there someone who is especially targeted / excluded / discriminated against?
	Where you live, would you say that everyone has the same access to health care?
Vulnerability	Do you feel that your basic needs in life are taken care of, and you are able to manage your health and wellbeing?
	What do you think makes people vulnerable to getting COVID-19?
Risk communication on COVID-19	Whom do you trust most for health advice?
	How did you learn about COVID-19 infection and pandemic rules? What has been your most reliable source of information? Is there something that could be done to provide better information on COVID-19 infection?
	What are your thoughts on pandemic regulations in Germany?
	Do you generally trust vaccines? Do you trust the current COVID vaccines?

The Vulnerability Assessment tool was translated into German by two researchers (VK, MC) whose native language is German. The translation was reviewed and finalised by four researchers and two external anthropologists at a meeting. The language of this translation was then simplified by a non-native German speaker (ZO). The tool was piloted with two international students (one Egyptian and one Turkish) who were not in the study population, as well as an Iraqi refugee who had lived in Germany for 20 years. Revisions were made in response to their feedback. The same researcher (ZO), who is a Turkish native speaker, translated the tool into Turkish, and provided examples from Turkish institutions and traditions to ensure cultural appropriateness. An Iranian field researcher who is a native Persian speaker and has a long-standing relationship with Afghan refugee families because of her volunteer and academic work translated the scale into Persian. She also included definitions for terms that the study population may have found unclear. In addition, because only this fieldworker conducted the interviews in Farsi, questions pertaining to cultural or institutional distinctions could be clarified.

Seven master’s students in public health and anthropology with experience in qualitative studies performed the interviews. Prior to data collection, training was conducted, starting with a discussion of the concept of vulnerability and continuing with role-playing interviews. Pilot interviews were conducted in tandem by one experienced and one less experienced interviewer. Solutions to the challenges and emotional burden of the interviews were discussed in weekly team meetings with the researchers. Eighteen interviews were conducted face-to-face, six were in refugee shelters, and seven were conducted online: nine in English, eight in Persian, seven in German and one in Turkish. The Persian was translated into English by the interviewer, the Turkish interview was coded by the Turkish researcher. The interviews were held in rooms where only the interviewer and interviewee were present.

The authors developed the questions for the experts based on the results of the second round of coding of the data from the first group. The interviews were conducted by one of the authors with the assistance of a fieldworker. The experts introduced themselves first, followed by a 10 min presentation on the vulnerability and resilience factors based on preliminary findings. Following their comments on the presentation, the experts were asked to explain how the pandemic has affected people of their concern and which support mechanisms have been ineffective. Participants representing an organization were asked which activities they could continue, and which activities were interrupted. After the experts’ comments on whether new resilience mechanisms have emerged, they were asked for their action recommendations. Additionally, each expert was asked questions specific to his/her field. The unclear issues that emerged in the analysis of the first group were also asked to be clarified. Three interviews were conducted at the experts’ workplaces and ten were conducted online.

#### 2.1.3. Data analysis

Data analysis largely followed the same protocol employed by the other country members in the study. Fieldworkers transcribed their own interviews and expert interviews. Transcripts were coded using the content analysis approach based on Mayring and Fenzl (20) in a three-round procedure. After all transcripts of the first group were read by two researchers (ZÖ and VK) separately to generate initial open codes, a consensus on selective codes was created. Following the independent coding of four interviews, the level of agreement between the different coders was evaluated, and divergences were discussed. Seven codes were excluded from the coding list, as they were not directly related answering the research question. In the second round of coding, one researcher read all the references under the codes, revised the code list, and identified sub-codes as positive and negative situations about each different aspect of the subject represented in the code. For example, in the neighbourhood code, proximity to nature was a sub-code associated with a positive situation, whereas the location of refugee shelters was a sub-code associated with a negative situation. Following a consensus meeting, all sub-codes were categorized to correspond to various vulnerability and resilience factors. The first draft of these factors was presented in expert interviews to ensure their validity and completeness. The feedback received led to minor adjustments of the characterisations of vulnerability and resilience factors in a consensus meeting. In the coding third round, expert interviews were coded using the same code manual and grouped to correspond to vulnerability and resilience factors. In a subsequent meeting, 12 categories reflecting vulnerability and eight categories reflecting resilience were selected to form a basis for action recommendations. These vulnerability and resilience factors were then grouped under three broad domains: “COVID-19 Prevention”; “Human Rights, Living and Housing Environment”; “Social Support.” The process ended with the validation check by one researcher for the consistency among codes, vulnerability and resilience factors, and quotes. Two native German speakers (VK, MC) translated the selected German codes into English, and a native Turkish speaker (ZO) translated the Turkish codes into English. Because all the Persian interviews had already been translated into English, there was no need to translate the quotes. All coding processes were carried out by the qualitative data analysis software NVivo (Release 1.6.2) (QSR International 2022).

### 2.2. Phase-2: formulation and discussion of action recommendations

Based on the vulnerability and resilience factors in addition to the expert recommendations defined in Phase-1, the researchers articulated a series of actions to support immigrants living in precariousness during and after the COVID-19 pandemic and similar crises. They compiled these action recommendations into a written document and then shared with participants and experts who had suggested actionable solutions in Phase-1, as well local government actors. In three rounds, the drafted recommendations were discussed and adapted according to their perceived relevance and feasibility, resulting in a set of recommendations that was supported by study participants with migration background, experts, and local government actors. Each round consisted of discussion and revision with a different group of community stakeholders to ensure recommendations were inclusive and attentive to the various, at times entangled, aspects of vulnerability.

In the first round, six experts from Phase-1, including a psychiatrist with many migrant patients and a person representing migrants with disabilities participated in a half-day panel that took place online. The experts assessed the action recommendations, which, in a draft report, had been shared with them before the online event, for their key characteristics (clarity, relevance to problem solving, action orientation, and potential to improve equality) as suggested in the relevant literature (21, 22). The recommendations were modified by the authors according to the panel discussion. The second meeting was a focus group discussion with twelve members of two migrant associations (Association of Migrant Women and Home of Solidarity). Each recommendation was rated on a Likert scale using the same criteria as the panel and each participant explained why he/she scored an item. Following this explanation, the group collectively determined how the item should be amended. The recommendation list was finalised after the third meeting with eight members of the Migration Council of Munich city, which consists of approximately 30 elected members. The final list was sent back to all panel and discussion participants to get their feedback.

### 2.3. Ethics

The study was approved by the Ethics Committee of the Medical Faculty of the LMU Munich as the competent approval authority (reference number 21-0244). Written informed consent was obtained from all participants. The methodology and findings are presented in a way that does not reveal the identity of the migrant participants. All experts stated that there was no need to conceal their identity, although care was taken not to provide a complete identification.

## 3. Results

### 3.1. Phase-1: challenges and resilience factors of people in precariousness during the COVID-19 pandemic

Twelve participants identified themselves as female, twelve as male and one as non-gender confirming (Table 3). Nine people had come from their home country to begin university in Munich and three of the thirteen refugees continuing their education. Seven refugees were currently living in a shelter. Eight of the experts had knowledge on the economy and poverty, five on labour rights, four on legal issues and four on healthcare (Table 1). The content analysis of the data yielded twelve vulnerability and eight resilience factors under three domains (Table 4).

**Table 3:** Characteristics of the study population – 1 (People living at the intersection of precarity and migration).

Person (P)	Age, gender, country of origin, years spent in Germany	Explanations
P-1	29, female, Colombia, 2	Masters student, lives with her husband. Poor German skills and hypothyroidism increase her isolation.
P-2	26, male, India, 1.5	Masters student, without German, lives in a student dorm. Lockdown caused psychological issues.
P-3	26, male, South Korea, 1.5	Masters student, without German, lives in a student dorm. Financial insecurity and pandemics have prolonged depression.
P-4	23, male, Turkey, 1	Bachelors student, feels financial insecurity. The pandemic has prevented him from working and learning German.
P-5	28, female, Eritrea, 1.5	Masters student, without German. Isolation affects her health and education.
P-6	26, male, Syria, 5	Bachelors student and refugee, the pandemic cost him his job and the library where he studied.
P-7	25, male, Pakistan, 1	Masters student without German who struggles with isolation, housing insecurity, and stress.
P-8	25, male, Syria, 5	Bachelors student and refugee. After losing his job due to the pandemic, he cannot pay rent and must borrow money.
P-9	36, female, Georgia, 14	Masters student with husband and 7 years-old daughter. She works two part-time jobs. Her husband lost his job, and their residence permit is insecure.
P-10	27, female, Iran, 1	Masters student without German. The embargo prevents bank transfers. Her pandemic-expired visa prevented her from visiting Iran. Stress caused her illness.
P-11	27, female, Afghanistan, 5	She lives in a shelter with her husband and two children.
P-12	68, female, Afghanistan, 2	She lives in a shelter with her husband and adult son. She suffers from back pain. She is illiterate.
P-13	47, male, Afghanistan, 2	He lives with his parents in a shelter. He has bone deformation which decreases the volume of his lungs.
P-14	36, female, Afghanistan, 2	She lives with her husband in a shelter. She lost her job due to the pandemic. She has a brain cyst.
P-15	36, male, Afghanistan, 3	He lives in a shelter and works as a tiler.
P-16	20, female, Afghanistan, 2	Her father abused his family after losing his job. She moved into a hostel with refugee aid.
P-17	32, female, Afghanistan, 3	Communication issues delayed her tuberculosis diagnosis. She lives with her husband and three children in a shelter.
P-18	35, male, Syria, 4	He is a refugee counsellor and himself a refugee.
P-19	32, male, Somalia, 3	He lives with his wife in a shelter. He is in a wheel-chair due to a gunshot and waiting for rehabilitation.
P-20	30, gender non-confirming, Uganda, 3	The person is registered in a shelter, but he/she lives with friends. She/he feels the stress of living in Germany.
P-21	28, male, Afghanistan, 2.5	He lives in a shelter. He has post-traumatic stress disorder.
P-22	25, female, Kenya, 7	She is taking care of older adults. She has post-traumatic stress disorder.
P-23	47, female, Turkey, 20	Divorced after losing her only child. She lives with social support.
P-24	24, female, Bulgaria, 4	Medical student. She feels to be discriminated because of her accent and she has financial insecurity.
P-25	Male, Greece, 3	He has a mild cognitive disability and lives alone.

**Table 4:** Challenges and resilience factors under three domains with quotes from the participants.

Domain	Vulnerability- (VF) and Resilience Factors (RF)
COVID-19 prevention	VF-1. Access to COVID-19 information
	VF-2. Compliance with hygiene measures
	VF-3. Access to COVID-19 test and vaccination
	RF-1. Networks and communities as information multipliers
Human rights, living and housing environment	VF-4. Increased racism, stigmatisation, and discrimination
	VF-5. Elimination of opportunities and environments for integration and socialisation
	VF-6. Deterioration of housing conditions
	VF-7. Living conditions and quarantine in refugee shelters
	VF-8. Accommodation problems of students
	VF-9. Violation of seasonal workers’ right to accommodation and healthcare
	RF-2. Equality by law
	RF-3. Organized struggle and solidarity against racism, stigmatisation, and discrimination
	RF-4. Proximity to green spaces
Social support	VF-10. Loss of the balancing influence of the school and child-care support
	VF-11. Discontinuation of support systems, despite increased need
	VF-12. Limited access to official institutions
	RF-5. Educational support for disadvantaged groups
	RF-6. Support by IT infrastructure
	RF-7. New volunteer workers
	RF-8. New and alternative support offerings

#### 3.1.1. Domain A. Covid-19 prevention

##### 3.1.1.1. Vulnerability factor 1. Access to COVID-19 information

For students and refugees with higher levels of education, accessing information on COVID-19 was not difficult as almost all followed English-language sources, but a the pandemic rules changed almost weekly, there was a great deal of concern about inadvertently violating them. Moreover, limited internet access in shelters made it difficult to keep track of the rules. For migrants with low levels of education, the inability to access and understand the information was a major problem. Common German-language information channels such as television did not reach them at all. In refugee shelters, notification was generally limited to the posting of informational leaflets. Aside from the participants who noted that these were available in their mother tongue, three refugees (P-11, P-13, P-14) said that the leaflets were only in German and therefore incomprehensible, while one woman (P-12) added that she was illiterate. Two experts explained that leaflets do not ensure health education, so practices such as hand washing should be demonstrated where people live, and solutions should be found together on their application under challenging conditions.

They should have used in shelters an accessible language, sharing videos, they should have shown how to wash hands, how to wear a mask, being there for them, not leaving them alone. (P-18)

Lack of access to information was accompanied by exposure to fake news and a certain distrust in the government, and conspiracy theories spread quickly in closed communities such as among refugee populations.

##### 3.1.1.2. Vulnerability factor 2. Compliance with (individual or personal) hygiene measures

No serious problems regarding access to protective equipment were reported, and those living in refugee shelters were provided with masks and disinfectants. However, two participants (P-15, P-17) explained that it was not possible to wear masks or disinfect hands in precarious working conditions.

You can’t work in construction with a mask, we keep the mask ready in our neck, and if someone comes to check, we put in front of their nose. (P-15)

Health problems that made it difficult to wear a mask were often cited (for example with those living with asthma), and, perhaps even more dramatic, wearing mask also made communication with others more difficult for people with poor or non-existent German language skills.

##### 3.1.1.3. Vulnerability factor 3. Access to COVID-19- testing and vaccination

Participants noted that routine testing was not practiced in refugee shelters and even those with symptoms went to the supermarket to get a quick test. The cost of rapid testing was also a concern; for example, students repeatedly asked for free testing at a student counselling centre. Not knowing how to apply for the COVID-19 vaccine, not being aware of being in the priority group, and not having access to IT equipment for application were common reported problems. Three female refugees (P-11, P-14, P-17) asked the interviewer’s opinion about the safety of the COVID-19 vaccine. One participant (P-18), a refugee counsellor, explained that mistrust of vaccinations stemmed from the way German authorities managed the quarantine process.

As guinea pigs … Asylum seekers wanted to be vaccinated but they didn't want to be the first. Being left alone in quarantine and police control destroyed their trust. (P-18)

Two experts explained that seasonal workers who do not have insurance were not entitled to vaccination, while another expert criticized Germany’s use of a national discourse in the vaccination campaign.

Looking at the advertising on the subway, "Germany is rolling up its sleeves," so that's already one of those, there's already talk about a national community. It paints a strange picture of solidarity in a society that is the opposite of solidarity. I don't need the government telling us to band together and support one another while blocking vaccine patents from being distributed to the Third World. E-10

##### 3.1.1.4. Resilience factor 1. Networks and communities as information multipliers

Migrant associations collaborated with other nongovernmental organizations to ensure that seasonal workers had access to COVID-19 vaccines. In some communities, information exchange took place within informal settings. Leaders in communities facilitated the access to vaccines, and information was shared through private communication channels.

Many people in the Turkish community had no idea what this meant or how to solve it operationally; "how do I solve this, where do I go, do I have to go to the family doctor?" … that was our problem until we discovered "there's a vaccination centre there, and you have to register accordingly" … And my wife was busy, setting up appointments for many people and acting as a multiplier so that they could all visit this vaccination centre. They had no idea a vaccination centre existed! (E-2)

#### 3.1.2. Domain B. Human rights, living and housing environment

##### 3.1.2.1. Vulnerability factor 4. Increased racism, stigmatisation, and discrimination

Both groups of participants explained that the pandemic reinforced racism and discrimination through various dynamics. The first was the belief that Asians caused the emergence and spread of COVID-19. Two experts and an Asian student explained that it had become common to be verbally insulted because of this. Another myth mentioned by participants was that migrants did not want to follow infection control rules or get vaccinated. It was explained that groups such as refugees, seasonal workers, etc. frequently could not access vaccinations while the precarious living and working conditions made contraction of COVID-19 infections more likely – reinforcing this myth, which in turn led to more visible discriminatory behaviour in society and official institutions.

At the supermarket my seven years old daughter forgot to keep the distance and got close to a German man. The man got very angry and shouted at her. My little girl was very upset and scared. (P-11)

According to experts, the press issued statements blaming specific groups, some politicians attributed migrants for the spread of the pandemic, a number of landlords refused to rent their homes to foreigners, human rights violations against seasonal workers could be ignored, and mosques were the first to close and the last to reopen during the pandemic, while churches remained open for an extended period. One of the experts mentioned that an increase in the number of applications to the counselling centre for victims of discrimination was an indication of this. Another factor cited as a trigger for fascism was the economic and social crisis caused by the pandemic.

In other cases, migrants are always labelled as criminals. This structural racism has become abundantly clear … People are always looking for someone to point the finger at. People with migration histories are always more likely to be identified as the criminals of other issues in society. We are to blame for unemployment. We brought the pandemic. … Right-wing extremist circles and lateral thinkers often intersect or overlap. (E-4)

##### 3.1.2.2. Vulnerability factor 5. Elimination of opportunities and environments for integration and socialisation

Venues that previously had encouraged people to socialise were closed during the pandemic. The impact of this was two-dimensional. The first was the weakening of people’s ties and interaction with their own communities, or even the lack of opportunity for such a community to form. For many, people from the same country constitute community. Refugee women considered other women in the shelter regardless of their country of origin to be their community; students referred to friends in the same dormitory as community, and some reported attending the same church or mosque as community. Students missed the times when they cooked together and helped each other, refugee women missed having tea together, and religious people missed worshiping with their congregation. This lack negatively impacted their resilience.

Going to the mosque is not just the religious thing, you can meet new people, your friends. … it creates togetherness in a new city, you don't feel alone as much as you once did. (P-7)

The second dimension was integration into the society. All activities such as festivals, courses organized for integration could not continue. With the interruption of language education, the feeling of foreignness was far from being resolved. The students spent all their time alone in their small rooms.

##### 3.1.2.3. Vulnerability factor 6. Deterioration of housing conditions

Housing was mentioned as one of the major problems in Munich since before the pandemic. Despite high prices, it is generally extremely difficult to find housing, especially for people without a German surname. Apartments are too cramped and often in poor condition to live in. During the pandemic, home office workers, children studying online, people with disabilities and psychiatric problems being at home all day were subject to housing shortcomings as serious sources of stress. Those who spent a significant portion of their monthly income on rent faced a financial crisis when they lost their jobs or were placed on short time working allowance.

If I have temporary work at the same time as the pandemic, if I only have 67% of my previous net income, and then have to provide for my family … but I still have to make sure that the rent is paid on time every month. (E-2)

My colleagues, who live in two rooms of a high-rise building with their families, small children, were particularly burdened when working from home. (E-11)

One expert explained that the circle of unemployment, despair and living in tight spaces was one reason behind the increased incidents of domestic violence being reported during the pandemic. Experts who were psychiatrists and addiction counsellors explained that online counselling is impossible for many people due to language barriers, limited IT possibilities and cramped space at home.

##### 3.1.2.4. Vulnerability factor 7. Living conditions and quarantine in refugee shelters

When asylum seekers arrive in Bavaria, they are housed in so-called anchor centres and then placed in shelters which are called collective accommodation centres; in these, conditions are relatively more favourable than the anchor centre. An Afghan refugee who spent a year in an anchor centre explained how difficult it was not to be allowed to leave the centre and to cook his own food. Shelters (anchor centres and collective accommodation centres) are located on the outskirts of the city, isolated from other settlements, and surrounded by fences as described by some participants “in the middle of nowhere.” It was explained that while drinking water from the tap in Munich homes is safe, doing so in shelters necessitates first filtering and boiling the water. The courtyards of the shelters do not offer opportunities for active recreation and are mostly used by men. The floors are separated for single individuals and families. At least two families or 10–20 people use the same corridor, toilet, bathroom, and kitchen. These people are from different backgrounds in terms of country of origin, language, religion, social backgrounds, etc. An Afghan woman (P-17) complaining about sharing a washing machine with nine men from Africa and a highly educated man (P-21) stating that he had nothing in common with the other men in his accommodation are some examples of the stated problems.

Quarantine is like imprisonment. They quarantined all three containers. I had been in my room for 23 days. Just in my room … We were quarantined for another week simply because one of us became infected. … Imagine, two toilets with 20 people. I cleaned two times, after one day it was getting into the worst condition again. (P-21)

During the lockdown no one was allowed to leave the shelter, causing crowded conditions to worsen. Participants who had lived/are living in shelters and four experts explained that the conditions made the spread of infections and chain quarantines practically inevitable. It was explained that many people hid their symptoms since they did not want to be isolated or to feel the pressure that other people were quarantined because of them. The inability to utilise free time effectively and the lack of internet access led to a greater burden on the mental health particularly of young refugees. Due to the quarantine, one refugee (P-21) could not take the required German exam, and another (P-15) could not take the vocational training exam. Those who experienced lockdown and quarantine in shelters faced significant mental health burdens, with pre-existing problems such as post-traumatic syndrome worsening. It was mentioned that the fact that everyone had to eat the same food during the quarantine was inconvenient, but there was no food insecurity.

##### 3.1.2.5. Vulnerability factor 8. Accommodation problems of students

The student counsellor explained that Munich is a centre of attraction for students, but the right to study is hampered by the limited availability of dormitories and high rents of the private alternatives.

Okay, there could be elite universities in Munich. But when students come here from other countries, they do not have a house, they do not speak the language. When their job went with Corona, it means working more to finance the rent, with a worse job than before. That means less time for education. (E-10)

The student participants also complained about housing costs. One student (P-08) who lost his job had to borrow money to pay the rent, while another student (P-06) had to work two jobs. Students spoke about the lack of study space at home and the closure of libraries during the pandemic, which severely affected their education. For those living in dormitories, the pandemic meant being completely isolated in a small room.

##### 3.1.2.6. Vulnerability factor 9. Violation of seasonal workers’ right to accommodation and healthcare

Two experts described the accommodation problem of seasonal workers, mostly from Eastern European countries, during the pandemic. Domestic workers were thrown out on the streets by their employers, while those working in farms and construction sites were caught in their shelters, lost their jobs and income, and were quarantined for long periods. Some did not have health insurance and those with EU insurance did not know how to benefit from it and/or employers prevent workers from using their EU insurance.

Most of them live in accommodation centres on construction sites. Those who are working in home care could no longer communicate with the outside. … Many people are working illegally, begging, doing sex work, and they have no health rights. (E-7)

##### 3.1.2.7. Resilience factor 2. Equality by law

Three participants (P-02, P-18, P-22) explained that although stigmatisation plays a role in everyday life, German law guarantees that all people are treated equally, and which is why they felt safe to some extent even during the pandemic.

People working in institutions like police, may have their personal beliefs … maybe they're homophobic or racist. But the law protects everyone. (P-20)

##### 3.1.2.8. Resilience factor 3. Organized struggle and solidarity against racism and discrimination

According to the experts, solidarity organizations responded to discriminatory speech and actions by actively intervening and assisting those affected. The organizations worked to ensure that seasonal workers who were left on the streets by their employers were given shelter and allowed to return home. They also brought attention to the poor living conditions of refugees in shelters as well as human rights violations. In addition, organized reactions and press statements were made against discriminatory statements in the press, but these were not sufficiently covered.

We make sure that the reporting is not discriminating … the statements of Minister X, who said that the Romanian workers had brought the virus. We wrote a statement, 20 associations signed it, but the press did not report on it. Minister X apologized for the statement that simply slipped out of his mouth. It doesn't just slip out, but from deep-seated prejudices. (E-8)

##### 3.1.2.9. Resilience factor 4. Proximity to green spaces and nature

Many participants named the green spaces or proximity to nature in Munich as an important resilience factor during the COVID 19 pandemic.

We are so integrated into nature here. … That's also a plus when it comes to Covid times. (P-4)

#### 3.1.3. Domain C. Social support

##### 3.1.3.1. Vulnerability factor 10. Loss of the balancing influence of the school and childcare support

Experts explained that the equalising effect of schooling had disappeared. The inability of non-German-speaking parents to support their children with their studies, the unsuitability of living spaces for online education and the lack of IT infrastructure had also further exacerbated existing inequalities.

If I consider the school system, the pandemic has revealed how racist our educational system is. Students who already faced numerous disadvantages prior to the pandemic suffered even more. Things where they compensated for certain problems, such as going to tutoring, where they were just able to keep up, have also faded, as have all compensatory means … everyday racism has been transferred from real life to digital life, because there was no everyday life there anymore. (E-4)

Not being able to attend school or day-care centres not only hindered children’s cognitive and social development, but also increased the childcare burden on families, especially mothers and single parents. A mother of a seven-year-old daughter (P-9) explained that she had given up on her master’s thesis and had not even sought medical attention due to the burden of childcare.

It's exhausting going to the doctor with her. Imagine me going to the gynaecologist with my daughter. … I do the bare minimum. (P-9)

##### 3.1.3.2. Vulnerability factor 11. Discontinuation of support systems, despite increased need

Lacking shelter, money, food and social connections, people were left alone when they needed organized support the most, experts said. Social workers were not allowed to enter refugee shelters or, in some cases according to some experts, preferred not to enter for fear of becoming infected. Support for needs such as household goods could not be provided, legal counselling could only continue online and at a limited level.

There were hundreds of people at the integration fair, either organizations, facilities or migrants would meet each other. All these opportunities have disappeared. (E-5)

The disruption of support systems placed a heavy burden on mental health. Four participants (P-2, P-3, P-18, P-21) described how the unmet mental health needs increased with the pandemic, with many people experiencing serious psychiatric problems. Support for problems such as addiction and post-traumatic syndrome almost ceased completely. Two refugees (P-18, P-21) stopped seeing a psychiatrist because they did not want to continue online. The addiction counsellor explained that it was impossible to conduct therapy online due to overcrowded housing and language issues. The statements of participants and experts also reflected that a significant part of the migrants needed support to navigate the health system. The consequence of not having this was the abandonment of the right to healthcare. For those who could access a health institution, language was a barrier because they could not be accompanied by a companion due to precautions. Participants gave examples of people with COVID-19 symptoms who did not go to a health facility despite severe symptoms.

##### 3.1.3.3. Vulnerability factor 12. Limited access to official institutions

During the pandemic, the accessibility of official institutions was severely restricted. Counselling on legal issues could not be obtained, residence permits could not be extended, and applications for employment could not be made. The fact that labour contracts or education were dependent on residence permits increased precariousness.

People had trouble making a living because the offices didn't work or weren't ready for the lockdown. For example, the employment contract depends on a residence permit. If you don’t have a permit, there is no employment contract. What do you do if your residence period expires? People were completely insecure … especially in the foreigners' authorities, district offices, and district administrative authorities. It took them over a year to respond to me. (P-21)

Two participants (P-18, P-21) explained that refugees staying in shelters could apply to a doctor only after getting a sick note from the person in charge to be approved by the social-welfare office, and since this process had become even more burdensome, many people gave up seeking health care altogether.

##### 3.1.3.4. Resilience factor 5. Educational support for disadvantaged groups

Online, face-to-face, or blended courses were offered for children from disadvantaged families. Online language courses were also offered for adults, which provided an important alternative for people who could not attend face-to-face classes for various reasons. On the other hand, all these options could only reach a limited group.

My friends have set up an online tutoring portal. They want to reach children who are likely to drop out of school if they don't get support. (E-4)

##### 3.1.3.5. Resilience factor 6. Support by it infrastructure and end devices

Initiatives were launched to provide IT equipment to children from disadvantaged families. However, it was added that this did could only reach a certain group.

We received 400 laptops from a large housing cooperative to distribute to our children. (E-9)

##### 3.1.3.6. Resilience factor 7. New volunteer workers

Experts reported increased voluntary participation of professionals unable to work during lockdowns, such as flight attendants and students suffering from social isolation.

Now, volunteers in a youth organization are now installing new operating systems. (E-9)

##### 3.1.3.7. Resilience factor 8. New and alternative support offerings

Alternative support such as telephone counselling or online services was created to compensate the services that could not take place in person. People who were previously unable to attend face-to-face appointments were able to join the online courses.

We made online courses and coaching. We switched from workshops to individual coaching. The women who started attending the online German courses would never have learnt so quickly otherwise. (E-5)

We built platforms where people could get a part of this network and get support, we organized online/hybrid events. (E-7)

Experts explained that their organizations had made efforts to reach people of concern, offering support in areas such as food and shelter that they had not previously provided.

Access to health services, vaccinations were given through the associations. There were a lot of help to the seasonal workers so that they don't lose their rights here. We provided them with many formalities. There were actions by associations that cooperated and put food in front of their door. (E-8)

### 3.2. Phase-2: action recommendations

Based on the vulnerability and resilience factors identified in Phase-1 of the study, the research team defined 24 action recommendations, organized into the different domains described above. This draft list was further developed in three rounds of discussion with experts, people with migration backgrounds, and local government representatives. In all three discussion and adaption rounds, these community stakeholders recognized that the structural nature of the mechanisms that can make migrants precarious require multifaceted action that target embedded marginalizing factors.

In the first round, which was an online expert panel, the participants suggested and formulated two new recommendations to be added based on the discussion: increasing the capacity and accessibility of psychosocial support facilities and empowering teachers to support their students psychosocially.

The revised recommendation list consisting of 26 items was discussed in the second round, an in-person focus group discussion with members of the Association of Migrant Women and the Home of Solidarity. The participants suggested that the problems of seasonal workers and refugees should be handled separately from each other, and employers should be strictly monitored in terms of workers’ contracts, insurance, housing and working conditions. While participants agreed migrants might need assistance with finding housing, they also emphasized the importance of migrants living together with the whole society to avoid the risk of ghettoization. Therefore, they suggested that migrants should first be supported to adapt to society, and then an integrated life could be considered.

It was suggested that the housing problem can only be solved through social models. Therefore, the term “affordable housing” used in the recommendations was changed to “social housing.” Participants also noted that solidarity should not be confused with the duties of the state. It was stated that society needs to learn how solidarity can be initiated and practiced. Strategies like neighbourhood solidarity networks and international kitchens initiated by city governments have been suggested as ways to address this. It was pointed out that the provision of services to migrants by health workers from the same country as themselves would lead to the emergence of a multi-class system, and the main solution to communication problems is language training.

Empowerment strategies for migrant women in terms of access to care and mechanisms to provide support for mental health were suggested. As a result, the number of recommendations increased to 29, with two new recommendations and one recommendation split into two items.

The third round, the meeting with the Migration Council focussed on the definitions of migration and precariousness. The participants explained that the socio-economic problems of people who were born and educated in Germany should be considered within the social class, not migration. News items that lead to stigmatisation in the media were emphasized, as well as awareness that not only the content but also the format of the news is critical. It was stated that migrants do not differ from non-migrants in complying with protective measures including vaccinations, and these facts should be proactively covered in the media. The number of recommendations increased to 30 after the revision. When the final list was sent to all participants, there were no suggestions for changes other than wording. Tables 5–7 present the final version of the action recommendations in three domains.

**Table 5:** Action recommendations under “COVID-19 Prevention” and related factors of vulnerability and resiliency.

Action Recommendation	Related vulnerability- and resilience factor	Status*
Communication of health information tailored to specific populations, including guidance on what to do if COVID-19 symptoms appear	VF-1, VF-2, VF-3, RF-1	A
Monitoring and clarifying of fake news	VF-1, RF-3	B1, B2, B3
Providing free protective equipment in risky working and living conditions	VF-2, VF-7, VF-9, RF-1, RF-2	A
Providing free and regular testing in risky working and living conditions	VF-3, VF-7, VF-9, RF-1, RF-2	A
Providing seasonal workers and asylum-seekers with easily accessible immunization services, such as mobile immunization clinics on-site	VF3, VF-7, VF-9, RF-1, RF-2, RF-8	B1

*Status: A, not modified; B1, modified in expert panel; B2, modified in focus group discussion; B3, modified in meeting with migration council.

**Table 6:** Action recommendations under “Human Rights, Housing, and Surroundings” and related factors of vulnerability and resiliency.

Action Recommendation	Related Vulnerability- and Resilience Factor	Status*
Strengthening all activities to combat racism, discrimination, and stigmatisation	VF-4, RF-2, RF-3	A
Preventing media allegations that link a disease to a specific group and rectifying government / media statements that violate their ethical obligations	VF4, RF-2, RF-3	B2, B3
Media dissemination of scientific facts that refute prejudices and rumours about certain groups	VF-4	C3
Raising society’s awareness of the living situations and challenges of different groups	VF-4, VF-6, VF-7, VF-8, VF-9	C1
Establishing social housing forms that support health and psychosocial well-being, including innovative solutions for students	VF-5, VF-6, VF-8, VF-9; RF-2, RF-4	B2
Planning neighbourhoods in a way that supports inclusion rather than ghettoizing	VF-4, VF-5, VF-6, VF-7, VF-8	C2
Establishing housing that neither isolates refugees from society nor exposes them to outbreaks	VF-4, VF-7, RF-2	A
Ensuring that seasonal workers are adequately housed in terms of health and human dignity and monitoring employers in this respect	VF-9, RF-2	B2
Providing emergency shelter for people who lost their accommodation due to crises	VF-8, VF-9, RF-8	B3
Control of employers in relation to employees’ rights and working conditions	VF-2, VF-9, VF-12, RF-2	A
Providing basic care and COVID-19 treatment for people without health insurance	VF-9, 1VF-2; RF-2, RF-8	B1

*Status: A, not modified; B1, modified in expert panel; B2, modified in focus group discussion; B3, modified in meeting with migration council; C1, emerged in expert panel; C2, emerged in focus group discussion; C3, emerged in meeting with migration council.

**Table 7:** Action recommendations under “Social Support” and related factors of vulnerability and resiliency.

Action recommendation	Related vulnerability- and resilience factor	Status*
Strengthening solidarity initiatives, publicizing good practices, emphasizing supplementing rather than replacing welfare state functions	VF-5, VF- 11, RF-5, RF-6, RF-7, RF-8	B2
Organizing activities that promote socialisation between groups	VF-5, VF-11	B2
Creating hybrid (online+ live) options solutions for integration in crisis situations	VF-5, VF-11, VF-12, RF-5, RF-8	A
Adapting quarantine rules so that support for the social needs of certain groups is not interrupted	VF-5, VF-6, VF-7, VF-9	A
Expansion of psychosocial services for specific groups such as students, teachers, single parents	VF-5, VF-11, VF-12	B1, B2
Educational support, especially for the disadvantaged students, during and after the crises	VF-10, RF-5, RF-8	A
Supporting teachers about students’ social challenges	VF-6, VF-10, RF-5	C1
Providing all students with IT equipment via affordable materials, without financial responsibility for damages	VF-10, RF-5, RF-6, RF-7	
Expansion of internet access	VF-10, RF-6	A
Extending childcare support to a larger group	VF-6, VF-10	A
Provision of children’s areas in health facilities in compliance with COVID-19 rules	VF-10	C2
Empowerment of migrants in terms of exercising their health-care rights	VF-9, VF-11	A
Providing support staff to overcome the language barrier in different settings (on-site, telephone counselling, written communication)	VF-11, VF-12	B2, B3
Raising awareness on support opportunities and reducing bureaucratic obstacles	VF-11, VF-12	B1

*Status: A, not modified; B1, modified in expert panel; B2, modified in focus group discussion; B3, modified in meeting with migration council; C1, emerged in expert panel; C2, emerged in focus group discussion.

## 4. Discussion

This study, conducted in Munich, revealed the vulnerability factors experienced by people living at the intersection of precariousness and migration during the COVID-19 pandemic. The findings indicate that the drawbacks of being a migrant often limited access to preventative measures and healthcare, and the living conditions in refugee centres, particularly so-called anchor centres, made outbreaks near-inevitable. The findings also demonstrated that the insecurity regarding legal status, employment, and housing, as well as the stigmatisation and discrimination that migrants experience, have exacerbated their sense of precariousness. On top of this, social support has weakened, and resilience mechanisms have not been sufficient to fill the gap. The circumstances that refugees encountered in one of Europe’s wealthiest cities emphasize the need for migrant-inclusive approaches to be employed to increase migrants’ resilience to upcoming crises (23). Recommendations were created in the study with the participation of experts and migrants to put the lessons learned from this pandemic into action for upcoming crises.

### 4.1. Intersectionality of vulnerability factors

The vulnerability factors we identified were grouped into three domains: (1) protection from COVID-19 infection, (2) human rights and living conditions, and (3) social support. These dimensions interact, and each factor within the dimensions triggered others. Publications on the pandemic’s effects (7, 24–29) confirm this intersectionality, which is caused by the fact that all these factors stem from the same underlying causes. As Siller and Aydın (30) pointed out, vulnerability is the product of structures that create adversity for marginalized groups. Multiple, simultaneous, and intertwined mechanisms of vulnerability are created by historically intersecting layers of discrimination, racism, and inequitable distribution of economic and social power (30). This situation, as identified in this study and in other publications (7, 24–29, 31), leads to a variety of mechanisms of vulnerability including limited access to health information and services due to language barriers and stigmatisation; inability to avoid the infection due to living conditions; lack of resources to cope with economic, and psychosocial impacts; unawareness of the rights one is entitled to; and failure to integrate in host communities. The resilience factors identified in this study compensated some vulnerability, albeit to a small extent. Our findings supported Siller and Aydin who argued that resilience is not the opposite of vulnerability, and that reducing vulnerability is as an integral part of increasing resilience (30).

### 4.2. Protection from COVID-19 infection

The first domain of action recommendation developed in Munich focused on the prevention of COVID-19. The effects of language difficulties—a well-known barrier to accessing health education and care— became increasingly noticeable during the pandemic (24, 27, 31, 32). However, as our research as well as COVID-19 outbreaks among migrants in Göttingen, another German city, have shown, information leaflets and translation in the mother tongue may not be enough to ensure access the information (32). Zimmermann et al., the authors of the Göttingen study (32), proposed more comprehensive information methods, as well as taking exposure to fake news and experience of distrust of authorities into account, which were also highlighted in our study. Our recommendations are highly relevant to these facts, but not as detailed as other guidelines (31, 33, 34) because they were not developed with specialized experts in risk communication.

Further, more research is needed to adapt health education to individuals that differ not only in terms of command of the German language but also regarding other social factors, as well as to individuals who face intersecting layers of discrimination. In line with studies carried out on the health and wellbeing of migrants in the COVID-19 epidemic, our research showed that language barriers create further limitations to the acquisition of adequate information about the public health situation (35–37). Another finding our research had in common with the Göttingen study was the expressed concern or mistrust of pandemic rules. This can stem from a deficiency in the employment of participatory approaches, as Zimmermann et al. explained (32). The effectiveness of participation has been demonstrated in two villages in China where radical quarantine measures were largely accepted (38). Poor previous experiences and low satisfaction in a healthcare or other institutional setting is a common barrier to the participation of migrants (39). The fact that we identified networks and communities as a resilience factor supports the importance of participation. For migrants unfamiliar with the health system, residing in a foreign country during a global pandemic with an unfamiliar health system when becoming ill can be expected to be particularly challenging. Being more isolated from traditional support networks such as friends and families may also have led to feelings of loneliness (39, 40).

Our participants mentioned no significant problems regarding access to personal protective equipment (PPE), which is a commonly reported issue in other places (26, 27). However, as IOM (27) reported, PPE’s applicability is limited by working and living conditions. Therefore, simply providing free PPE is insufficient to ensure prevention of disease outbreaks; living and working conditions that prohibit or inhibit its use must also be taken into account. Even though, as stated in numerous papers (24, 27) and echoed in our study, navigating a foreign health system and access to registration can be difficult for migrants, our study indicated a lack of targeted programs for early treatment and vaccination, as well as a lack of routine testing for those in high-risk settings. In addition to low levels of health literacy and language skills, stigmatisation was a significant barrier to accessing preventive measures and health services as also reported by WHO and IOM (27, 31, 41). Abuses by employers are compounded to these problems for seasonal workers. This facts shows that the international regulations such as EU Charter of Fundamental Rights and the International Convention on the Protection of the Rights of All Migrant Workers and Members of their Families and equality by law in Germany, reported by participants as a factor of resistance, are not sufficient to ensure that everyone has access to healthcare and preventive services related to COVID-19 (29). As outlined in our recommendations and the WHO’s assessment tool for refugee and migrant health in the context of COVID-19 pandemic, specific programs for migrants are needed (33). Furthermore, targeted interventions are required to improve migrant workers’ rights, workplace health and safety knowledge, and access to healthcare (42).

### 4.3. Human rights, living conditions, and social support

Stigmatisation and scapegoating of migrants, hate speech and xenophobic incidents were widespread throughout the pandemic (26, 27, 42). Although public leaders supposedly have political, social, and legal responsibilities to oppose pandemic-linked xenophobia and discrimination (1, 42), even elected politicians in Germany made disparaging remarks about migrant workers as explained by the experts. Therefore, we recommend actions based on zero-tolerance policy for racism and hate speech, regardless of its source. Furthermore, as stated by Abubakar et al. (42), the work of fact-checking, truth promotion, and vociferous objections should not be left solely to migrants and their advocates. Our recommendations include increasing public awareness of different groups’ living conditions and difficulties, which is supported by Twigg et al. (24), who emphasized the importance of developing positive and evidence-based narratives that fully account for migrants’ suffering and positive contributions to their societies.

The effects of structural racism and other systems that cause injustice were clearly visible in our findings regarding living conditions, which made migrants more vulnerable to contracting COVID-19 and increased their social isolation (27, 28, 30, 32, 43). The most severe circumstances were seen in the shelters for refugees and seasonal workers. Our recommendations for providing suitable housing for seasonal workers and refugees as well as shelter for those who have lost their homes due to crises are consistent with other guidelines (28, 33). However, housing in Munich has become the epicenter of the problem, which was already a challenge for low-income migrants including students. WHO (33) advised to governments to help migrants to access affordable and adequate housing. However, our participants stated that “affordability” could not prevent the commodification of the right to housing, and “social housing” should be defended instead of “affordable ones.” This is supported by Patuzzi (43) who stated that challenges migrants face in housing markets have their roots in protracted public underinvestment in social housing, rather than integration barriers alone. Additionally, migrants should be a partner in neighbourhood planning in order to implement suggestions like preventing ghettoization and enhancing access to green spaces, which we identified as a resilience factor.

Migrants in precarious situations require support with basic needs such as food and water (28, 33). In Munich, where safe tap water is almost everywhere available, respondents living in shelters complained about the poor quality of water. The fact that food security concerns was not expressed could be attributed to our questions. Expert indicated that there was a problem with food support, and NGOs were unable to address this gap due to a lack of resources or the lockdown. As a result, food, water, and financial support should be included to the action recommendations. Since language was identified as significant barriers to needs access, stronger language support, particularly in healthcare, was suggested as in other recommendations in the literature (27, 33, 44). Another recommendation was to remove bureaucratic barriers to healthcare, social services, and immigration procedures. However, similar to other world regions, the suspension of immigration processes left many migrants unsure of their legal status and vulnerable to the pandemic’s effects (27, 33, 44). As many countries, Germany have adopted flexible procedures during the pandemic (24). However, given the intensity of the mentioned challenges, it is necessary to monitor how effective these solutions are and whether institutions are implementing them.

Pervasive digitalization of integration, education and other services risks further marginalizing the migrants, who lack digital skills, technology, and language competency (43). This was also the case in our study. However, the experts claimed that online alternatives were crucial during the pandemic notwithstanding their limitations. Although they agreed that informal, low-stress interactions may be harder to reproduce in the digital space (43), according to the experts, online alternatives made it feasible to offer integration and language classes for adults, one-to-one tuition for students and organizations could have solidarity gatherings with a bigger participation. As a result, hybrid solutions during crisis were recommended. Another related recommendation is to improve access to IT-technology and connectivity. The city of Milan successfully implemented this recommendation, which is also included in the WHO Assessment Tool (33, 34). On the other side, even if barriers to access to technology are reduced, the educational gap between children has grown significantly (24, 26, 30, 33). Schools or other formal educational systems provides children with positive and nurturing relationships, which Herbers et al. (45) referred to as adaptive systems. The study’s recommendation was to compensate for the interruption in this adaptive system during and after crisis.

As a result of the mentioned reasons the mental health of migrants, which was already high due to their precariousness and pre-existing psychiatric issues, worsened dramatically during the pandemic. This fact is also well documented in the literature (26, 36). Our study demonstrated that, despite the increasing demand for psychosocial support, even the pre-existing assistance was disturbed by the pandemic. In accordance with WHO, the expansion of community mental health support was recommended by our participants, as well as improving teachers’ abilities in supporting their students with the social challenges.

### 4.4. Solidarity or responsibilities of social state

According to our research, during the pandemic, NGOs and volunteers in solidarity organizations attempted to fill gaps in public service by facilitating access to vaccination, providing counselling services, ensuring food, accommodation, and healthcare for migrant workers, as is done in many countries (26, 42). Such initiatives have been proposed as a recommendation as in the WHO Assessment Tool (33). Our participants’ warning, on the other hand, that volunteerism and solidarity cannot replace the social state is critical. Patuzzi (43) also stated that volunteerism should not be viewed as a silver bullet for integration as doing so carries significant risks. One is that it can become an excuse to cut public budgets. Another risk is that volunteers often lack the specialized skills and knowledge. Patuzzi also stated that initiatives born of neighbourly solidarity can serve as a springboard for more structured, long-term engagement if city authorities take steps to support them, emphasizing the importance of public authority (43).

### 4.5. Empowerment of migrants

Previous reports have highlighted the importance of co-creating solutions and public health responses with migrants (24, 29, 31, 34, 43). Accordingly, recommendations were developed with migrants based on the issues raised by them during the first phase of the study. Community stakeholders participated in the meetings recognized that the structural nature of the vulnerability mechanisms require multifaceted action that target embedded marginalizing factors. As previously stated, these factors often have historical and political roots that necessitate coordinated action at the governmental level; however, the inclusion of key people and civil society actors is equally important if participatory policy is to have an impact, particularly on those who frequently fall outside the reach of policy efforts and impact. Such participatory and inclusive approaches, in which the very people affected by intersectional vulnerability factors are involved in formulating and incorporating policy solutions that address these factors, are a type of community engagement that can be especially effective because it integrates the perspectives and experiences of those who stand to benefit the most from it (46). Moreover, diversifying audiences and producers of public involvement does not only make it more probable to engender direct effects related to the specific issues at stake, it also helps to effectuate a shift to community-led engagement, organized action and policy making that can have diminishing effects on exclusionary and marginalizing mechanisms overall (47, 48). As such, involving communities can help to level power relationships between different stakeholders, including government actors, community organizations and researchers; to develop capacity, access, and agency among community members; and to strengthen community connections by purposefully targeting commonly shared issues, creating and sustaining meaningful collaborations (49). However, a critical issue is how to ensure that policymakers incorporate evidence from participatory approaches for action into their agenda. Our strength in this regard is that the stakeholders who participated in our research owned the recommendations. Members of the city’s other councils, for example, heard the recommendations from the Migration Council. However, participation in meetings does not necessarily mean involvement in decision-making for disadvantaged groups as long as there is a huge power imbalance between them and decision-makers (50). Participation should be transformed into a means of enabling marginalized groups to overcome the barriers and empowering them, not helping to maintain elite institutional hegemony (50). Therefore, the recommendation of our participants regarding women’s empowerment, as highlighted by WHO (33), should be the guiding principle.

### 4.6. Limitations

This study provides a wide perspective framework on the challenges faced by migrants during the COVID-19 pandemic. This broad approach, however, came at a cost of thoroughly examining each vulnerability and resilience factor in depth. Furthermore, the recommendations developed are limited to the vulnerability and resilience factors identified in the local context in the city of Munich. When compared to international body guidelines, they must be more actionable and comprehensive. However, it is expected that recommendations developed with the community will differ in terms of content and technical specifics from guidelines developed by specialists. Violations of seasonal workers’ human rights that have been a problem in Germany during the epidemic (24, 28) could only be seen from an expert’s perspective because it was not possible to conduct interviews with workers. The effects of precarious employment for groups other than seasonal workers could not be addressed. More focused research is required to fully comprehend these factors and challenges unique to various migrant groups. Even though the study was conducted from the perspective of a global network, the study’s reliance on data from a single city limit the study’s generalizability. The recommendations developed using Munich as an example can be viewed as a starting point for addressing structural inequality and collectively preparing for crises in other global cities.

### 4.7. Conclusion

According to the study’s findings, migrants in precarious situations, such as refugees, seasonal workers, and students with financial difficulties faced significant challenges during the pandemic. Migrants met the crisis when they were already in a crisis. Therefore, the first set of recommendations developed in this study, such as a more targeted and effective approach to racism, aim to reduce structural vulnerabilities that feed into precariousness and that exacerbate migrant and refugee vulnerabilities. Tailored interventions, rather than blanket approaches for the entire society, form a second group of recommendations. The third group is aimed at increasing social resilience, which includes improved access to educational and psychosocial support as well as empowerment of migrants. However, while efforts should be collaborative and require the expertise and on-the-ground knowledge and networks of community actors, governments should be the primary source of these efforts, rather than migrants and their advocates. The paths authorities choose with respect to migrant-inclusive COVID-19 response-and-recovery efforts will shape societies’ levels of risk to future crises (24). The significant parallels between actions that should be taken for the pandemics and the climate issue (51–53) demonstrates the pressing need for this agenda and provides apertures for a shared approach.

## Data availability statement

The raw data supporting the conclusions of this article will be made available by the authors, without undue reservation.

## Ethics statement

The studies involving human participants were reviewed and approved by Ethics Committee of the Medical Faculty of the LMU Munich (reference number 21-0244). The patients/participants provided their written informed consent to participate in this study. Written informed consent was obtained from the individual(s) for the publication of any potentially identifiable images or data included in this article.

## Author contributions

ZÖ, MG, A-MV, and MC contributed to the study’s conception and design. ZÖ, MC, and A-MV supervised the interview process. ZÖ, AB, and VK conducted expert interviews. The participant meetings were organized and moderated by ZÖ, AB, VK, MG, and MC. ZÖ, AB, and VK analysed the data, while MC validated the consistency of the codes, vulnerability and resilience factors, and quotations. ZÖ drafted the initial version of the manuscript. MG and MC wrote sections of the manuscript. All authors contributed to the revision of the manuscript, read it, and approved the final version.

## Funding

The Sonar-Global Network was supported by the European Union’s Horizon 2020 research and innovation programme under grant agreement No. 825671.

## Conflict of interest

The authors declare that the research was conducted in the absence of any commercial or financial relationships that could be construed as a potential conflict of interest.

## Publisher’s note

All claims expressed in this article are solely those of the authors and do not necessarily represent those of their affiliated organizations, or those of the publisher, the editors and the reviewers. Any product that may be evaluated in this article, or claim that may be made by its manufacturer, is not guaranteed or endorsed by the publisher.
